# ID 107 - Delandistrogeno Moxeparvoveque para o Tratamento da Distrofia Muscular de Duchenne

**DOI:** 10.5327/2237-9622.2025.v34s1.107

**Published:** 2025-11-25

**Authors:** Cristiane de Cássia Bergamaschi, Mariana Del Grossi Moura, Jéssica Cumpian, Luciane Cruz Lopes, Juliana Machado Rugolo, Viviane Del Lama Cardoso Salas, Aline do Nascimento, Silvio Barberato-Filho

**Keywords:** delandistrogeno moxeparvoveque, distrofia muscular de duchenne, monitoramento de horizonte tecnológico

## Abstract

**Introdução:**

Distrofia muscular de Duchenne (DMD) é a forma mais comum e grave de distrofia muscular causada por mutações no gene que codifica a distrofina. A ausência ou deficiência da proteína distrofina leva a danos repetidos nas fibras musculares e desencadeia inflamação crônica, fibrose progressiva e comprometimento das células-tronco musculares. Este alerta de monitoramento de horizonte tecnológico foi elaborado com a finalidade de informar quanto aos potenciais impactos de delandistrogeno moxeparvoveque (Elevidys®) para o tratamento da DMD.

**Método:**

A estratégia de busca foi realizada em duas etapas, em 15 de abril de 2024, e não houve restrição quanto ao idioma. Na primeira etapa, foi realizada a busca nas bases de dados Cortellis, da Clarivate Analytics, e de registros de ensaios clínicos na plataforma ClinicalTrials.gov. Na segunda etapa, identificaram-se ensaios clínicos publicados por meio de busca nas bases de dados MEDLINE, Embase e Cochrane Library. Os títulos recuperados foram inseridos no gerenciador de referências EndnoteTM, para remoção de duplicatas e triagem.

**Resultados:**

Oito registros de ensaios clínicos e quatro ensaios clínicos publicados foram incluídos. Dos registros, seis ensaios clínicos estavam em andamento e dois concluídos. Sete deles foram conduzidos nos Estados Unidos e um na Espanha. Quatro estudos correspondiam à fase I ou II (n=67 participantes); dois estudos à fase III (n=520) e dois estudos à fase IV (n=648). Cinco estudos avaliaram em específico a população pediátrica (n=677). Os achados de eficácia e segurança dos quatro ensaios clínicos revelaram mudança da linha de base na quantificação de expressão da proteína distrofina delandistrogeno moxeparvoveque; avaliações funcionais com melhoria sustentada ao longo de um ano e estabilização da função motora em até dois anos após o tratamento; e eventos adversos considerados leves e moderados, resolvidos nos primeiros 90 dias após a infusão. O evento adverso mais frequente foi vômito e foram relatados como eventos adversos graves rabdomiólise, aumento nas enzimas hepáticas e necessidade de hospitalização.

**Conclusão:**

O perfil de segurança e eficácia de delandistrogeno moxeparvoveque para o tratamento de DMD apresenta-se consistente entre os estudos. No entanto, o número pequeno de estudos e de pacientes avaliados, torna o poder de recomendação fraco. Estudos de fase 3 controlados por placebo e com maior tamanho de amostra e de tempo de seguimento dos participantes são necessários para validar de forma significativa os achados de eficácia e de segurança a longo prazo.

